# Merging Visible Light Communications and Smart Lighting: A Prototype with Integrated Dimming for Energy-Efficient Indoor Environments and Beyond

**DOI:** 10.3390/s25196046

**Published:** 2025-10-01

**Authors:** Cătălin Beguni, Eduard Zadobrischi, Alin-Mihai Căilean

**Affiliations:** 1 Department of Computers, Electronics and Automation, Stefan cel Mare University of Suceava, 720229 Suceava, Romania; catalin.beguni@usm.ro (C.B.); eduard.zadobrischi@usm.ro (E.Z.); 2 Integrated Center for Research, Development and Innovation in Advanced Materials, Nanotechnologies and Distributed Systems for Fabrication and Control, Stefan cel Mare University of Suceava, 720229 Suceava, Romania

**Keywords:** adaptive illumination, adaptive lighting, energy efficiency, intelligent lighting infrastructure, light dimming, user centered lighting, smart lighting, sustainable building technologies, visible light communications

## Abstract

This article proposes an improved Visible Light Communication (VLC) solution that, besides the indoor lighting and data transfer, offers an energy-efficient alternative for modern workspaces. Unlike Light-Fidelity (LiFi), designed for high-speed data communication, VLC primarily targets applications where fast data rates are not essential. The developed prototype ensures reliable communication under variable lighting conditions, addressing low-speed requirements such as test bench monitoring, occupancy detection, remote commands, logging or access control. Although the tested data rate was limited to 100 kb/s with a Bit Error Rate (BER) below 10^−7^, the key innovation is the light dimming dynamic adaptation. Therefore, the system self-adjusts the LED duty cycle between 10% and 90%, based on natural or artificial ambient light, to maintain a minimum illuminance of 300 lx at the workspace level. Additionally, this work includes a scalability analysis through simulations conducted in an office scenario with up to six users. The results show that the system can adjust the lighting level and maintain the connectivity according to users’ presence, significantly reducing energy consumption without compromising visual comfort or communication performance. With this light intensity regulation algorithm, the proposed solution demonstrates real potential for implementation in smart indoor environments focused on sustainability and connectivity.

## 1. Introduction

In the context of the global climate crisis and increasing pressures on energy resources, the buildings sector is at the center of an unprecedented systemic transformation. Responsible for around 30% of final energy consumption [1] and over a third of global CO_2_ emissions, buildings [2], from hospitals and universities to homes and public institutions, are increasingly becoming not just consumers but also active actors in the transition towards sustainability [3,4,5]. The technological revolution triggered by LED lighting [6] and, more recently, the emergence of Visible Light Communications (VLC) [7,8] and Light-Fidelity (Li-Fi) [9,10] technologies, open up new perspectives in energy efficiency, connectivity and smart space management [11,12].

In parallel, ambitious European Union (EU) policies, such as the European Green Deal [13] and the recast Energy Performance of Buildings Directive (EPBD) [14], impose strict energy performance standards and massively stimulate the renovation of inefficient buildings. This convergence between technology, regulation and the need for decarbonization is accelerating the transition to a smart, digitalized and climate-neutral built ecosystem, where every watt saved counts and every building becomes an active link in a decentralized and sustainable energy grid.

The buildings sector is a central pillar of global energy consumption. According to the International Energy Agency (IEA) [15,16], this share has continued to grow, reaching a share of electricity of 35% in the energy mix of buildings in 2022, compared to 30% in 2010. In the EU, buildings absorb about 40% of final energy, of which 80% is used for heating [17], cooling and hot water, and around 10% is directed to lighting [18]. However, this sector offers huge potential for efficiency, especially through modern technologies such as LEDs and intelligent adaptive lighting systems [19,20]. Replacing conventional light bulbs with efficient LEDs, combined with daylight harvesting and automated dimming functions, can reduce lighting consumption by up to 90% [21]. At a European scale, this transition alone could save 38 TWh annually, equivalent to the energy consumption of over two million households. Despite this potential, progress has been slow: between 2014 and 2019, total consumption in buildings stagnated, largely due to the low renovation rate, below 1% per year [22]. To address these challenges, the EU has adopted a series of ambitious directives and initiatives under the European Green Deal. Within this framework, the EU aims to achieve climate neutrality by 2050 and a minimum 55% reduction in greenhouse gas emissions by 2030 [13]. The recast EPBD 2024 stipulates that, from 2028 for public buildings and 2030 for private buildings, all new construction must be nearly zero-emission buildings. In parallel, the annual renovation rate must reach between 2.5% and 3%. Complementary measures are introduced through the Energy Efficiency Directive (EED), alongside funding schemes such as Renovation Wave (EUR 210+ billion) and +100 Climate Neutral Cities (EUR 650 billion), focused on transforming the built stock.

Institutions such as hospitals, universities and airports are a priority in these interventions, with high energy requirements and intense traffic. For example, ENERPOS, a university building on the French island of La Réunion, proves the applicability of the “net-zero” model in a tropical climate, where only 14% of consumption is allocated to lighting [23]. Hospitals, with a requirement of 300–1000 lux, implement individually controlled LEDs and Building Management System (BMS) technology, generating savings of 20–60% and improving visual comfort by 10–15% [24]. Universities use presence and brightness sensors for optimization, and in the real estate sector, buildings certified in energy classes A–B obtain 5–10% higher rents and access green financing such as those offered by KfW (Germany), through the “Effizienzhaus” standard [25].

The transition to LEDs, combined with automation, VLC and BMS, is radically transforming the role of buildings in the urban ecosystem: from passive consumers, they become smart, green and user-centric platforms. These technologies reduce emissions, optimize comfort, improve safety and create notable economic benefits, especially in the real estate sector. Beyond energy savings, they redefine built infrastructure as sustainable digital hubs, essential for the cities of the future. Given that the buildings sector is responsible for 40% of final energy consumption and 36% of energy-related CO_2_ emissions, action in this area is essential [26]. Moreover, as around 75% of European buildings are energy inefficient and the deep renovation rate is below 0.3% per year [27], there is an urgent need to accelerate large-scale renovation efforts to improve energy performance, reduce emissions, and align with the EU’s climate neutrality goals. In this context, the Renovation Wave aims to renovate at least 35 million buildings by 2030, mobilizing over EUR 275 billion in annual investment [28]. In addition to climate measures, the Green Deal includes the “Zero Pollution Action Plan”, aiming to reduce energy-related air pollution by 55% by 2040 and fully decarbonize lighting and heating systems. Fiscal measures such as the extension of the EU Emissions Trading Scheme (ETS) to the buildings sector will boost the transition to clean energy solutions. The recast EPBD 2024 requires that, from 2030, all new buildings must be “Zero Emissions Buildings” (ZEB), powered exclusively by local renewable sources. Joint Research Centre (JRC) estimates indicate a potential to reduce consumption by over 14% by 2030 and prevent 60 MtCO_2_/year equivalent to Hungary’s emissions in 2019 [29]. The “Fit for 55” package supports these efforts with policies and funds totaling over €700 billion by 2030, targeting buildings, mobility and digitalization. It is estimated that up to 1.8 million green jobs could be created in construction, energy efficiency, IT and renewables [30]. After 2030, ambitions increase: the EU economy aims for a net-zero structure by 2050, with renewables covering 85–95% of final consumption. JRC modeling shows that zero-emission buildings can reduce thermal energy demand by up to 50% and emissions by over 80% compared to 1990. Future innovations include LED lighting of over 200 lm/W, adaptive sensors, and VLC/Li-Fi. In parallel, new energy storage technologies, active façades and Building Integrated Photovoltaics (BIPV) will allow buildings to become prosumers, i.e., to consume, but also produce and inject energy into the grid and/or energy storage systems. Thus, by 2050, all public buildings will be required to actively participate in the flexibility of energy networks, becoming prosumers and sustainable digital hubs.

In this effort toward new solutions for improved energy efficiency, VLC technology emerges as a solution that combines energy efficient lighting and wireless communications [31,32]. VLC technology operates in the visible spectrum, with frequencies between 400 and 800 THz, radically different from the conventional radio frequencies used in Wi-Fi (2.4 GHz/5 GHz) or 5G (24–86 GHz) networks. This difference offers a major advantage in terms of spectral capacity, the visible spectrum is approximately 10,000 times wider than the licensed radio spectrum, allowing a significantly higher number of interference-free communication channels. Another important aspect is user density. VLC allows up to 16 users to be connected simultaneously per luminaire, without any significant drop in speed or signal quality [33]. In terms of energy consumption, VLC does not add any additional input: the system uses LED light pulses invisible to the human eye, generated within the same lighting infrastructure already installed [7].

In addition, VLC technology is immune to ElectroMagnetic Interference (EMI), which makes it ideal for critical spaces such as hospitals, controlled environments, research laboratories, aircrafts and military bases. Another key advantage is the physical security of the communication channel: the light signal cannot pass through walls, which eliminates the risk of data interception outside the room. This is essential in government, military or financial environments. In the updated vision of the EU on “smart-ready” and “zero-emission” buildings, VLC becomes a strategic technological pillar. Being an integral part of the lighting infrastructure, VLC systems can function as a digital backbone in smart buildings, without contributing to the energy load. Unlike Wi-Fi networks, which require separate routers and additional cabling, VLC directly uses LED sources to transmit information, thus eliminating infrastructure and maintenance costs. By integrating with BMS, VLC enables a redundant, decentralized and secure communication network, which ensures real-time control of environmental parameters—temperature, humidity, CO_2_ level, human presence, etc. VLC technology is optimal for high-density IoT applications, seamlessly connecting sensors, cameras, interactive panels, or mobile terminals, without generating interference or network congestion. In high-traffic areas such as universities, airports, hospitals, shopping malls or office buildings, where Wi-Fi networks quickly reach capacity limits, VLC adds a fast, reliable and scalable parallel network. In addition, using VLC in parallel with Wi-Fi or 5G allows for physical network slicing between critical (transmitted via light) and non-critical (transmitted via radio) applications, increasing the resilience of the entire system. Several leading technology companies and research centers have initiated VLC implementations in real environments. Signify (formerly Philips Lighting), through its TruLiFi platform, offers commercial light communication systems capable of reaching speeds of 250 Mbps for each connected user [34]. Oledcomm, a French leader in the Li-Fi field, has developed dedicated solutions for operating rooms, where RF signals are completely prohibited, guaranteeing sterile and interference-free communication [35]. The Fraunhofer Heinrich–Hertz Institut in Germany has tested VLC technology in medical environments, such as intensive care units, highlighting the ultra-secure and interference-free nature of light-based communication suitable for sensitive healthcare settings [36].

In parallel, Massachusetts Institute of Technology (MIT) and the University of Oxford have conducted tests on university campuses and research centers, demonstrating the use of VLC as a viable alternative to congested Wi-Fi networks [37,38,39,40]. Also, in train stations and airports, where latency plays a crucial role in signaling or ticketing systems, VLC provides fast and accurate transmissions without the risk of interference. In modern office buildings, VLC systems allow each individual office to be assigned a digital hotspot, using a simple LED light fixture. This granularity radically transforms the way data networks in commercial buildings are designed and operated. As buildings transition from being energy consumers to being digital prosumers and hubs, VLC supports this transformation with a unique set of energy, operational and environmental benefits. Studies conducted by MIT and the University of Oxford show that integrating digital communications directly into the lighting system reduces the total consumption of IT infrastructure by up to 20%, mainly due to the elimination of separate cabling, switches and energy-consuming network equipment. In addition, the fact that VLC uses existing LED infrastructure means a significant reduction in the material footprint—less equipment, fewer resources, fewer indirect emissions.

Another approach [41] demonstrates that it is practical to build glazing that both generates electricity (via luminescent solar concentrator photovoltaics) and supports visible-light communication. This could reduce the need for separate infrastructure for lighting and data while integrating energy production into façades. Devices like this are stepping stones for future buildings that are capable of communication and are also sustainable. The glazing can serve multiple roles, such as window, photovoltaic generator, and receiver, in a single element. This work shows that meeting building and photovoltaic standards, one of the major barriers to deployment of such advanced materials, is achievable. That removes a large hurdle to moving from lab research to commercial and architectural implementation. While the results are promising, there are trade-offs to consider, such as lower transparency versus higher energy capture, cost of quantum dots, long-term stability under harsh weather, and the cost of manufacturing large panels. The paper demonstrates a working proof that balances these trade-offs sufficiently to pass certification. In terms of performance, the potential of VLC and LiFi technologies is huge. Thus, existing studies indicate that optical wireless technologies could achieve data rates above 100 Gb/s [42]. Although at this moment this potential is only theoretical, preliminary experimental demonstrations shown data rates of few tens of Gb/s [43,44,45,46,47,48,49,50,51,52,53,54]. On the other hand, other studies have shown that VLC and LiFi technologies can effectively support multiple users and accommodate user mobility [44,45,46,47,48,49]. Another challenge in this field concerns the security of these emerging applications [50]. In addition, in the context in which the concept of integrated communication and sensing is emerging as the next great thing, the use of optical wireless communications for distance measurement and relative positioning is also highly promising [51]. Within this regard, recent studies have shown that centimeter accuracy positioning can be achieved in indoor scenarios [52,53].

Within this technological framework, VLC technology perfectly meets the requirements imposed by the European Green Deal, which foresees a digitalized, efficient and climate-neutral economy by 2050. In this sense, VLC contribute to the indirect reduction of CO_2_ emissions associated with IT and traditional networks, an aspect often overlooked in the carbon footprints of buildings. Moreover, in economic terms, the global VLC market is estimated to grow from USD 2.7 billion in 2025 to USD 7–27 billion by 2030, with a CAGR of 21% to 41% [54]. This dynamic makes VLC technology one of the most promising emerging markets in the smart and digital building ecosystem.

In a context where energy saving solutions are this important for human society, this article is proposing an improved indoor VLC solution capable to provide simultaneous data transfer and illumination. The article expands the basic preliminary results of [55], while demonstrating additional features and providing a scalability evaluation. On the VLC perspective, the prototype proved capable of providing data rates of up to 100 kb/s and BER lower than 10^−7^. Even if the data rate performance is below the existing state-of-the-art demonstrated by LiFi technology, the proposed concept is able to dynamically adjust the light dimming in order to maintain a proper illumination at the workspace level. Thus, the VLC transmitter can adapt the LED duty-cycle in the 10–90% range, reducing it when the workspace is sufficiently illuminated by natural light entering through the window or by artificial light coming from neighboring luminaires. Otherwise, the VLC transmitter is increasing the duty cycle to ensure an illuminance level of around 300 lx at the workspace level. Once the experimental performance is proven, the second part of this work focuses on demonstrating the energy efficiency and scalability of the concept. For this second purpose, a set of simulations are made in order to investigate the concept behavior in a given scenario. Thus, the investigation considers a working space for up to six users and aims to evaluate how the concept could adapt to the users’ presence in order to provide optimal lighting conditions, connectivity, and energy efficiency. The simulation results confirm the concept’s scalability and indicate potential for significant energy saving.

Therefore, the main contributions of this work are as follows:

- Development of a dual-function VLC prototype that simultaneously supports indoor illumination and data communication, with integrated dynamic dimming functionality based on ambient light conditions.
- Experimental validation of the VLC system’s performance, demonstrating reliable data transmission of 100 kb/s with a BER lower than 10^−7^, ensuring communication robustness even under variable lighting conditions.
- Implementation of an adaptive dimming algorithm that enables the VLC transmitter to adjust the duty cycle within a 10–90% range, effectively balancing energy consumption and visual comfort by responding in real time to natural and neighboring light sources.
- Simulation-based scalability analysis of the proposed VLC solution in a multi-user office-like environment, evaluating the system’s ability to maintain acceptable lighting levels (~300 lx) and connectivity for up to six users while minimizing energy consumption.
- Demonstration of energy-saving potential, with simulation results confirming that the proposed system can dynamically allocate resources based on users’ presence and illumination needs, thereby contributing to sustainable indoor lighting and communication infrastructure.

These contributions emphasize the practicability of incorporating VLC into smart lighting systems for energy-efficient, scalable, and communication-capable indoor environments, particularly relevant in contexts where sustainability and reliable connectivity are critical.

## 2. Model and Methods

### 2.1. General Assumptions

The performance of a V2V VLC links is significantly influenced by the amount of data-carrying light that reaches the surface of the VLC receiver’s photosensitive element. In addition to the influence of the VLC channel, this amount of light is largely determined by the distance between the transmitter and receiver. In indoor VLC environments, where lighting devices serve the dual purpose of illumination and data transmission, achieving a proper balance between lighting quality and signal modulation becomes crucial. The goal is to provide not only an efficient communication link but also a comfortable and effective visual environment. While irradiance can be easily controlled, doing so does not automatically ensure adequate illuminance for visual comfort. Human eyes are most sensitive to wavelengths around 555 nm, in the green portion of the visible spectrum, where luminous efficacy peaks. This visual sensitivity varies depending on ambient lighting conditions and the nature of the user’s activity. Therefore, both the optical transmission parameters and the required illuminance at the working surface must be optimized. To do so, the concept of luminous efficacy $K_{r}$ must be factored into the system’s design, which is defined as the ratio of one of the basic photometric quantities to its corresponding radiometric quantity as per the following formula [56]:

(1) $$K_{r}=\frac{\Phi_{v}}{\Phi_{e}}=\frac{I_{v}}{I_{e}}=\frac{E_{v}}{E_{e}}=\frac{L_{v}}{L_{e}},$$

where $\Phi_{v}$ is the luminous flux, $\Phi_{e}$ is the radiant flux (power), $I_{v}$ is the luminous intensity, $I_{e}$ is the radiant intensity, $E_{v}$ is the illuminance, $E_{e}$ is the irradiance, $L_{v}$ is the luminance, and $L_{e}$ is the radiance. The parameter of interest here is the illuminance, which can be written:

(2) $$E_{v}=K_{r}\cdot E_{e}.$$

**Figure 1:**
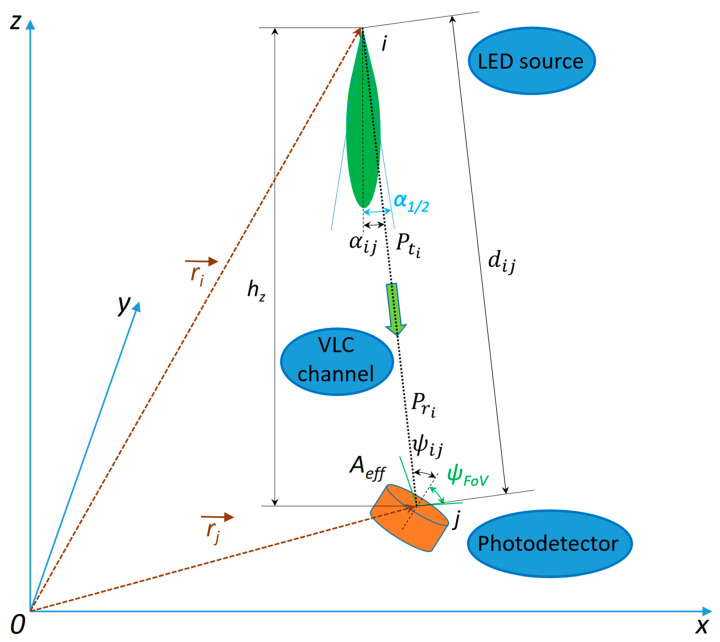
Diagram of light transmission from an LED source to a photodetector.

The defining relation for irradiance is:

(3) $$E_{e}=\frac{{d\Phi }_{e}}{ds},$$

where ${d\Phi }_{e}/ds$ is the infinitesimal element of radiant flux over the corresponding element of area in the surface. In the particular case of a photodetector (Figure 1), the irradiance will be:

(4) $$E_{e}=\frac{P_{r}}{A_{eff}}\;,$$

where $P_{r}$ is the received power at the detector level and $A_{eff}$ is the effective detection area. If the source is a generalized Lambertian emitter, the angular distribution of radiant flux is:

(5) $$R_{0}(\alpha )=\left(\frac{m+1}{2\pi }\right)\cdot {cos}^{m}\alpha .$$

Assuming that $\psi \leq \psi_{FoV}$, where $\psi_{FoV}$ is the Field-of-View (FoV) angle of the photodetector, then for a surface with effective area $A_{eff}$, situated at distance *d*, seen under angle *ψ*, the differential solid angle dΩ is:

(6) $$d\Omega =\frac{A_{eff}\cdot \cos\psi }{d^{2}}.$$

Therefore, the power emitted in this differential solid angle is given by:

(7) $$dP=I_{e}\left(\alpha \right)d\Omega =P_{t}\cdot R_{0}\left(\alpha \right)d\Omega .$$

This power is exactly the received power and, based on Equations (5)–(7), it can be written further on as:

(8) $$P_{r}=P_{t}\cdot R_{0}\left(\alpha \right)\cdot \frac{A_{eff}\cdot \cos\psi }{d^{2}}=P_{t}\cdot \left(\frac{m+1}{2\pi }\right)\cdot {cos}^{m}\alpha \cdot \frac{A_{eff}\cdot \cos\psi }{d^{2}}.$$

Using Equations (2), (4) and (8), the illuminance will be written as:

(9) $$E_{v}=\frac{(m+1)\cdot K_{r}\cdot P_{t}}{2\pi \cdot d^{2}}\cdot {cos}^{m}\alpha \cdot \cos\psi .$$

### 2.2. The Mathematical Model for VLC LED-Based Luminaires

In order to determine the efficiency of VLC LED-based luminaires, the scenario considered is a work place with the following assumptions and parameters:

- room dimensions are L × l × h = 8 m × 6 m × 3.5 m;
- the luminaires are 6 in total, placed in a 2 m × 2 m ceiling grid (regular spacing);
- each workspace is under one luminaire, at height $h_{d}=0.8\;m$ from the floor, so the vertical distance from each luminaire to its workspace is: $h_{z}=3.5\;m-0.8\;m=2.7\;m$
- transmitters are generalized Lambertian sources, i.e., each luminaire is constituted from multiple tiny LEDs, closed enough to each other to be considered a Lambertian source as a whole;
- maximum radiant power per each luminaire is $P_{t}=36\;W$;
- the required illuminance at the workspace level is $E_{req}=300\;lx$;
- each luminaire can transmit VLC data using VPPM with a duty cycle $\delta_{i}$ ranging from 1% to 99%, so each luminaire will have a transmitted power given by $P_{t_{i}}=\delta_{i}\cdot P_{t}$, where $\delta_{i}\in \left\{0.01\cdot k|k\in N,1\leq k\leq 99\right\}$;
- each luminaire has the same luminous efficacy, $K_{r}=150\;lm/W$;
- each luminaire is numbered with i, where i∈{1,2,…,6} and each workspace position is numbered with j, where j∈{1,2,…,6};
- the photodetector has a wide FoV, enough to capture ambient light from its surroundings;
- the half-power angle, $\alpha_{1/2}$, of the luminaire is 75°, and, in this case, the Lambertian coefficient is determined to be:
  (10) $$m=-\frac{ln2}{\ln\left(cos\alpha_{1/2}\right)}\approx 1/2.$$
- in this scenario, the photodetector is presumed to be oriented toward the ceiling, so α=ψ.

Based on Equation (9) and the upper-mentioned assumptions, the illuminance at workspace *j* will be determined by the contribution of each luminaire, noted with *i*, using the following equation:

(11) $$E_{v}^{\left(j\right)}(i)=\frac{(m+1)\cdot K_{r}\cdot {P_{t}}_{i}}{2\pi \cdot d_{ij}^{2}}\cdot {cos}^{m}\alpha_{ij}\cdot \cos\psi_{ij}.$$

The coordinates for the luminaires will be:

(12) $$\vec{r_{i}}=(x_{i},y_{i},h).$$

The coordinates for the workspace places will be:

(13) $$\vec{r_{j}}=(x_{j},y_{j},h_{d}).$$

Distance from luminaire *i* to workspace *j* is:

(14) $$\vec{d_{ij}}=\vec{r_{j}}-\vec{r_{i}}=(x_{j}-x_{i},y_{j}-y_{i},-h_{z}),$$

(15) $$d_{ij}=\left\|\vec{d_{ij}}\right\|=\sqrt{{\left(x_{j}-x_{i}\right)}^{2}+{\left(y_{j}-y_{i}\right)}^{2}+{h_{z}}^{2}.}$$

The emission angle, which is equal to the incidence angle ψ, will be determined based on the diagram of light transmission from Figure 1:

(16) $$\cos\;\alpha_{ij}=\frac{h_{z}}{d_{ij}.}$$

Based on the adjustable duty cycle, denoted by $\delta_{i}$, the corresponding $P_{t_{i}}$ value will be determined with the following relation:

(17) $$P_{t_{i}}=\delta_{i}\cdot P_{t}.$$

In this model, the duty cycle δ is treated as a control variable.

The final illuminance equation at workspace place j will be determined from Equations (11), (15)–(17) as:

(18) $$E_{v}^{\left(j\right)}=\sum_{i=1}^{6}\frac{\left(m+1\right)\cdot K_{r}\cdot P_{t}\cdot {h_{z}}^{m+1}}{2\pi \cdot {\left[{\left(x_{j}-x_{i}\right)}^{2}+{\left(y_{j}-y_{i}\right)}^{2}+{h_{z}}^{2}\right]}^{\frac{m+3}{2}}}\cdot \delta_{i}$$

Replacing *m* from Equation (10), the simplified relation will be:

(19) Evj=∑i=163·Kr·Pt·hz324π·xj−xi2+yj−yi2+hz27/4·δi

### 2.3. The Mathematical Model for Natural Light

In order to determine the influence of natural light, the scenario includes a window in the work room and the following assumptions and parameters will be taken into consideration:

- the window is placed on the left 6 m-wall, is 5 m wide, centered, and 1 m high, starting at 1 m from the floor, so the points on the window surface have the following coordinates: $x_{w}=0$, $y_{w}\in [0.5,5.5]$ and $z_{w}\in [1,2]$;
- the exterior natural light enters the room through the window clear double-glazed glass with a transmission factor $\tau_{G}=0.7$;
- the window is considered a rectangular Lambertian emitter as a diffuse luminous surface;
- the emission is normal to the window plane, along the x-direction (perpendicular to the 6 m-wall);
- exterior illuminance (in lux) is $E_{v,\;ext}\in$ {5000, 10,000, 20,000};
- the photodetector remains oriented toward the ceiling, so the incidence angles are calculated accordingly.

The window is regarded as a diffuse luminous surface of known area and known emitting illuminance, so the natural indoor emission illuminance will be:

(20) $$E_{v,\;win}=\tau_{G}\cdot E_{v,\;\;ext.}$$

Each point, having an infinitesimal area of $dA_{w}={dy}_{w}\cdot {dz}_{w}$, emits luminous flux that contributes to workspace j. Equation (9) can also be used in this case, so the illuminance received at point j from this patch can be written as:

(21) $${dE}_{v,win}^{\left(j\right)}=\frac{\left(m+1\right)\cdot K_{r}\cdot {P_{t}}_{w}}{2\pi \cdot d_{wj}^{2}}\cdot {cos}^{m}\alpha_{wj}\cdot \cos\psi_{wj}$$

Based on Equations (1) and (19):

(22) $$K_{r}\cdot {P_{t}}_{w}={d\Phi }_{v,win}=E_{v,win}\cdot dA_{w}=\tau_{G}\cdot E_{v,ext}\cdot {dy}_{w}\cdot {dz}_{w}.$$

In the end, the illuminance received at point j from the infinitesimal point is:

(23) $${dE}_{v,win}^{\left(j\right)}=\frac{\left(m+1\right)\cdot \tau_{G}\cdot E_{v,ext}}{2\pi }\cdot \frac{{cos}^{m}\alpha_{wj}\cdot \cos\psi_{wj}}{d_{wj}^{2}}{dy}_{w}{dz}_{w}.$$

Knowing that each infinitesimal window patch was considered as an ideal Lambertian emitter, then m=1. The FoV can be chosen large enough so that $\psi \leq \psi_{FoV}$ for all the window patches. Also, knowing that the photodetector is placed upwards, then the following relation is always true: αwj+ψwj=π2. With the upper-mentioned assumptions, a point on the surface of the window can be considered a Lambertian source, with the following coordinates:

(24) $$\vec{r_{w}}=(0,y_{w},z_{w}).$$

The vectorial distance from one point of the window to each workspace j will be determined with the following relation:

(25) $$\vec{d_{wj}}=\vec{r_{j}}-\vec{r_{w}}=(x_{j},y_{j}-y_{w},h_{d}-z_{w}),$$

(26) $$d_{wj}=\left\|\vec{d_{wj}}\right\|=\sqrt{{x_{j}}^{2}+{\left(y_{j}-y_{w}\right)}^{2}+{\left(h_{d}-z_{w}\right)}^{2}.}$$

**Figure 2:**
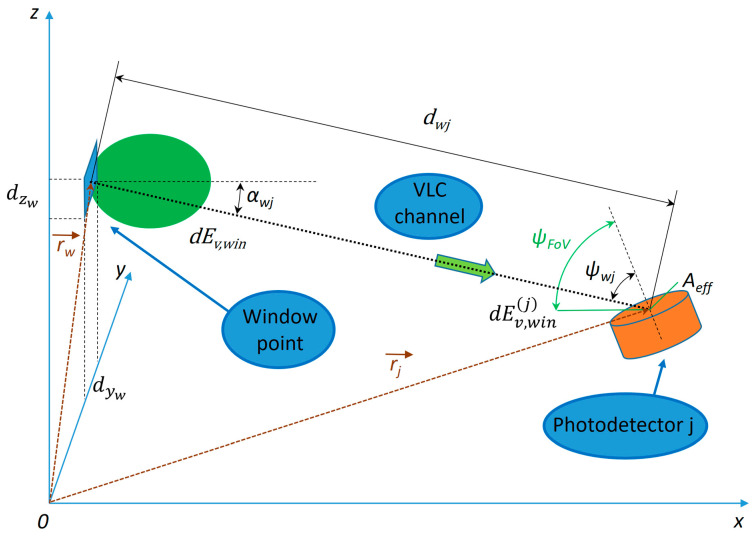
Diagram of light transmission from an infinitesimal window point to a photodetector.

Cosine of incidence and emission angles will be calculated based on Figure 2 with the following relations:

(27) $$\cos\;\alpha_{wj}=\frac{x_{j}}{d_{wj}.}$$

(28) $$\cos\;\psi_{wj}=\frac{z_{w}-h_{d}}{d_{wj}}.$$

Based on Equations (22), (26) and (27), the illuminance at workspace j from an infinitesimal point on the window can be written as:

(29) $${dE}_{v,win}^{\left(j\right)}=\frac{\tau_{G}\cdot E_{v,ext}}{\pi }\cdot \frac{x_{j}\cdot (z_{w}-h_{d})}{{\left[x_{j}^{2}+{\left(y_{j}-y_{w}\right)}^{2}+{\left(h_{d}-z_{w}\right)}^{2}\right]}^{2}}{dy}_{w}{dz}_{w}$$

To compute the total illuminance at workspace j, the contribution from all points on the window surface are integrated:

(30) $$E_{v,win}^{\left(j\right)}=\frac{\tau_{G}\cdot E_{v,ext}}{\pi }\int_{z_{w}=1}^{2}\int_{y_{w}=0.5}^{5.5}\frac{x_{j}\cdot (z_{w}-h_{d})}{{\left[x_{j}^{2}+{\left(y_{j}-y_{w}\right)}^{2}+{\left(h_{d}-z_{w}\right)}^{2}\right]}^{2}}{dy}_{w}{dz}_{w}$$

### 2.4. The Mathematical Model for Combined Illumination

Because the light sources do not emit coherently, they do not interfere. Therefore, the principle of superposition can be applied in order to determine the total combined illuminance at workspace j:

(31) Ev,totalj=Evj+Ev,winj=∑i=163·Kr·Pt·hz324π·xj−xi2+yj−yi2+hz274·δi+τG·Ev,  extπ∫zw=12∫yw=0.55.5xj·(zw−hd)xj2+(yj−yw)2+(hd−zw)22dywdzw

### 2.5. The Mathematical Model for the Energy Consumption Optimization

Each LED has a dimming level $\delta_{i}\in \left\{0.01\cdot k|k\in N,1\leq k\leq 99\right\}$, and its power consumption is directly proportional to $\delta_{i}$. So, the objective function is to minimize total electrical energy consumed by the luminaires, which is proportional to the total duty cycle of all VLC LED sources:

(32) $$F\left(\delta_{i}\right)=\underset{\delta_{1},\ldots ,\delta_{n}}{\min}\sum_{i=1}^{n}\delta_{i}$$

For each occupied desk without sufficient natural lighting (i.e., where natural light is less than 300 lux), the total illuminance at the desk (natural and artificial) must be at least 300 lx. So, for each user j∈{1,2,…,6}, the total illuminance at each workspace must meet or exceed the required visual threshold (i.e., 300 lx):

(33) $$E_{v,\;total}^{\left(j\right)}=E_{v}^{\left(j\right)}+E_{v,win}^{\left(j\right)}\geq E_{req},\;\forall j.$$

In order to determine the minimum energy consumption, a matrix A with the energy contributions is computed. Each element $A_{ij}$ represents how much illuminance at workspace j is contributed by luminaire i per unit dimming (δ=1):

(34) Aij=3·Kr·Pt·hz324π·xj−xi2+yj−yi2+hz27/4

Based on Equation (32), the following relation is also computed:

(35) $$A\cdot \delta +E_{v,win}^{\left(j\right)}\geq E_{req},\;\forall j.$$

**Figure 3:**
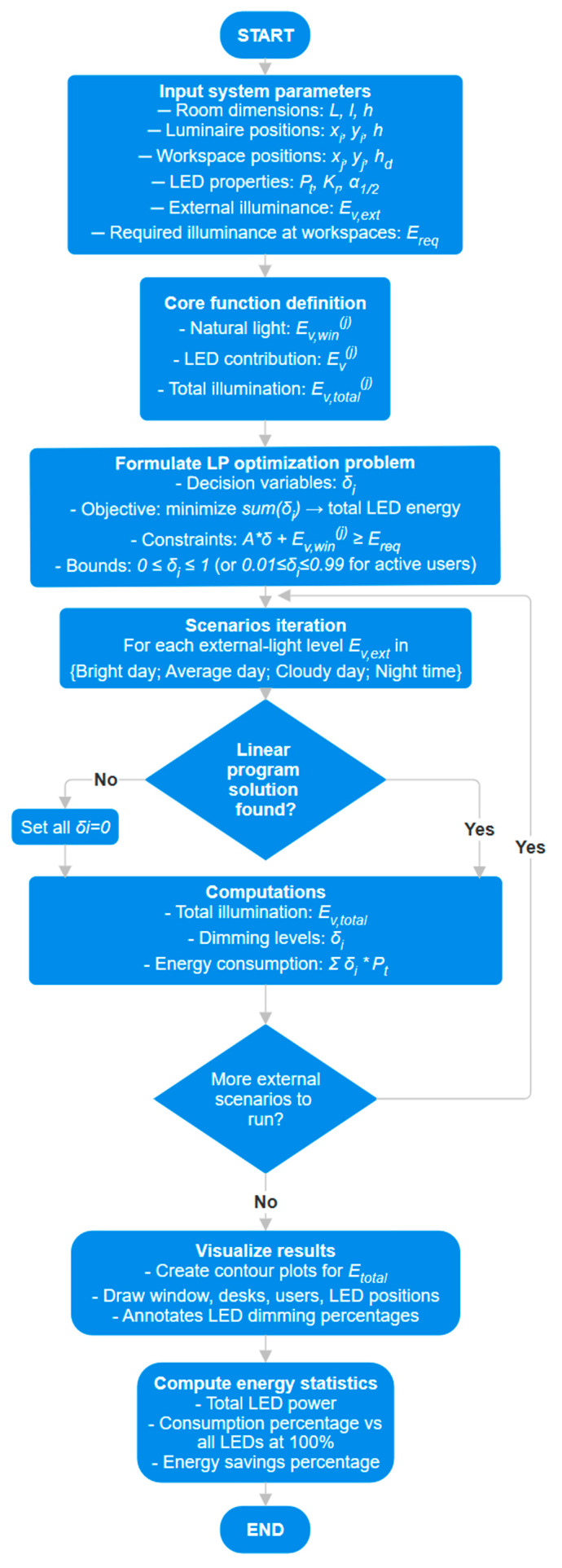
Flowchart of the implemented algorithm.

Figure 3 presents a comprehensive flowchart of the algorithm, illustrating all steps from input parameters to final outputs. It encompasses natural and artificial lighting, linear program optimization, and post-processing for minimum dimming.

## 3. Implementation of the Visible Light Communications Prototype and Experimental Evaluation

### 3.1. Hardware Implementation of the Visible Light Communications System

The focus on improving energy efficiency was initially through the adoption of LED technology. Currently, the interest has shifted towards smart solutions that integrate presence, motion, and ambient light sensors, enabling advanced functions that were previously unattainable with traditional lighting sources.

In this context, the current section aims to implement a preliminary scenario for the VLC prototype in the work room, under data transmission conditions, to evaluate energy efficiency. In this regard, the indoor VLC system is designed to continuously adjust its duty cycle based on the ambient illumination level from adjacent or external light sources. For reduced computational load, the duty cycle is adjusted between 10% and 90%, in 10% steps. However, as experimentally demonstrated in [57,58], light dimming can also be made with a 1% accuracy, enabling light dimming within 1–99%, or even with greater precision. Nevertheless, higher resolution dimming increases the processing frequency required to maintain the same data rate.

**Figure 4:**
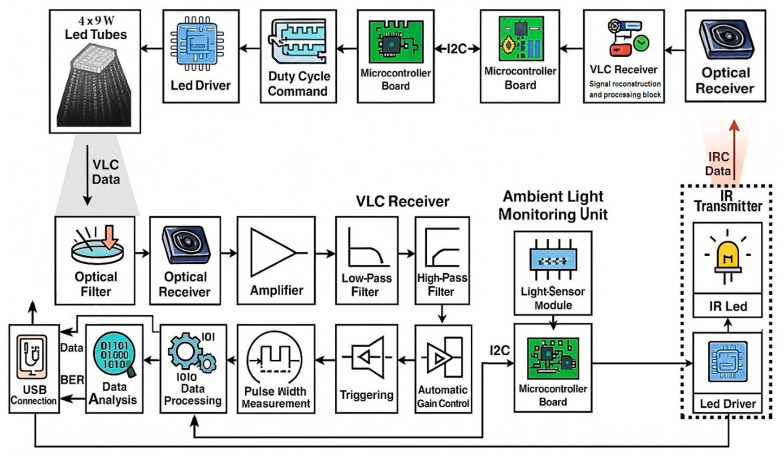
Schematic representation of the visible light communications system.

Figure 4 illustrates the proposed diagram of the indoor VLC system. The concept features an LED lighting infrastructure consisting of six standard luminaires, each originally designed to accommodate four 60 cm fluorescent tubes. For the purposes of the experimental scenario, one luminaire has all fluorescent tubes replaced with 9 W LED tubes. The luminaire integrates a VLC transmitter dedicated to the mobile receiver within its corresponding workspace. The light emitted by the LED tubes is modulated by a microcontroller with a 600 MHz ARM Cortex M7 processor through a driver circuit. To enable data transmission, the microcontroller sends initially a header, building a frame structure that includes the payload. The header contains synchronization pattern, duty cycle values, and other relevant parameters. This frame is then encoded and modulated for optical transmission. In order to implement light intensity control over data modulation, the VLC transmitter uses a modified Variable Pulse Position Modulation (VPPM) scheme [59]. VPPM is specifically designed for VLC applications, offering both high-precision dimming and simplified mechanism for data encoding [7,59]. Similarly to Pulse Width Modulation (PWM), VPPM allows light intensity to be adjusted by varying the pulse width. At the same time, data encoding is based on Pulse Position Modulation (PPM) principles. This modulation technique prevents flickering phenomena by ensuring equal light energy for both binary ‘1’ and ‘0’ bits.

The primary innovation of this concept is its capability to continuously and independently adjust the duty cycle of the transmitted VLC signal. This allows the system to maintain the desired illumination level without compromising data transmission, by responding to the light intensity measured in the workspace. To achieve this, the VLC infrastructure receives data from the infrared optical transmitter located at the workspace level and connected to the mobile VLC receiver, providing constant feedback on the workspace illumination, which is then used to regulate the transmitted VLC signal.

The VLC receiver is designed to collect various types of information required for applications that do not demand high data rates, and is also capable of transmitting data via the IR transmitter. To perform this efficiently, the device is fitted with an optical filter that rejects wavelengths outside the spectrum of interest, an optical receiver based on a photodiode connected to a transimpedance amplifier circuit, low-pass and high-pass filters, an Automatic Gain Control (AGC) amplifier, a Schmitt trigger for signal regeneration, and signal processing circuits for the acquired signal, based on a microcontroller with an ARM Cortex M7 processor, overclocked to 1.08 GHz.

In addition to the mobile VLC receiver and the infrared transmitter, the system also contains an ambient light sensor based on a photodiode connected in a voltage divider configuration, whose signal is sent to a microcontroller board. This device is capable of sensing the illumination level in the workspace area. The ambient light sensor captures light emitted by its dedicated VLC transmitter, lighting sources from other luminaires, and natural light entering through the window. The microcontroller then compares the measured illuminance values with the required optimal level and, based on the result, transmits via the InfraRed (IR) channel whether the VLC transmitter’s duty cycle should be adjusted. Therefore, it is imperative that all transmitters communicate with each other because adjusting the lighting in one workspace indirectly affects the other workspaces. Thus, when an increase in light intensity is requested in one area, the corresponding luminaire must be activated. However, this also affects the light reaching other areas, so their luminaires must reduce intensity according to the algorithm. This anticipatory compensation helps prevent lighting fluctuations that could otherwise trigger a chain reaction through the activation of individual feedback loops.

Table 1 presents the summary of testing parameters for the VLC prototype.

### 3.2. Experimental Testing Procedure and Experimental Results

The main purpose of these experiments is to validate an innovative functionality intended for VLC systems used in indoor spaces, namely, advanced optimization of energy consumption. Within these evaluations, the aim is to demonstrate that the prototype is able to: (i) dynamically adjust the VLC transmission mode to increase user comfort, while significantly improving energy resources efficiency, and (ii) ensure the continuity of the wireless connection.

**Figure 5:**
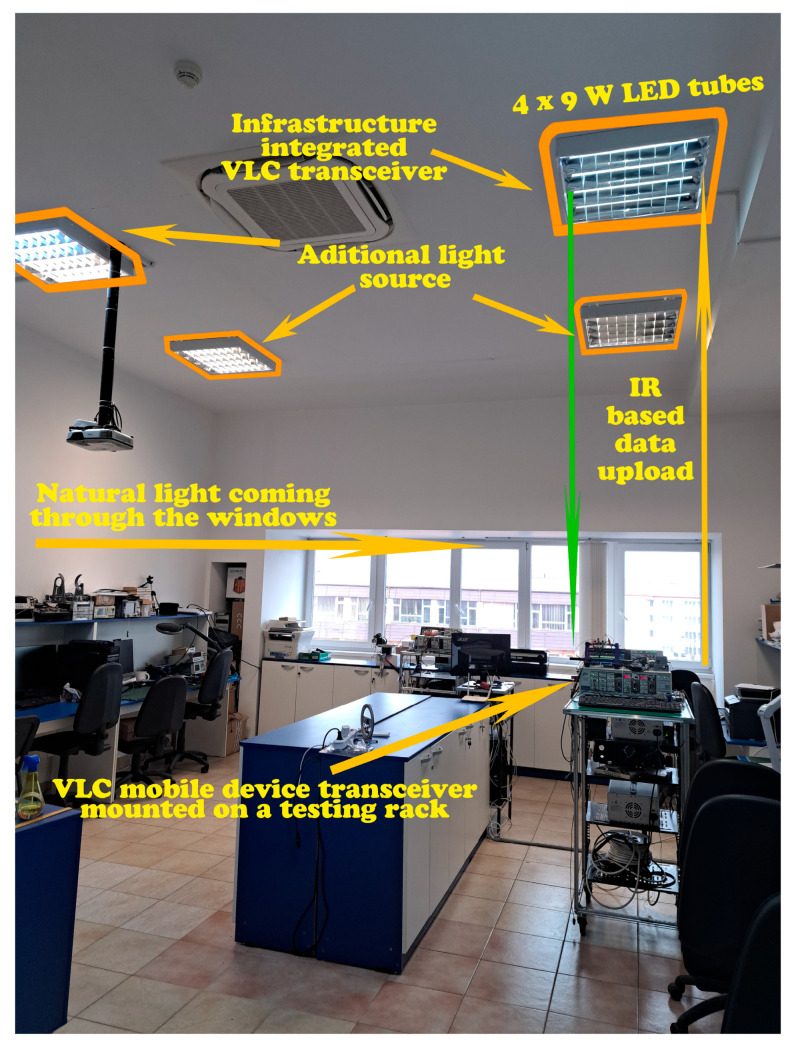
Experimental setup illustrating the VLC transceiver integrated into the indoor lighting system, along with the VLC-enabled mobile device.

As illustrated in Figure 5, the prototype is installed in a research laboratory configured as an open-space zone designed for six users, each having an individual lighting luminaire positioned above their activity area. The ceiling height of the room is 3.5 m, while the work surface is located at 80 cm from the floor. The tests were carried out under natural lighting conditions, during the day, with sunlight entering through the windows. To analyze the system’s ability to adapt to changing contexts, the experiment was conducted during a partly cloudy day, at different intervals and in contexts of fluctuating light intensity. Additional testing setups were also envisioned for circumstances where natural light was insufficient, being supplemented by artificial sources from adjacent areas occupied by other users. The purpose of these experimental scenarios is to examine whether the prototype has the ability to effectively calibrate itself in relation to ambient lighting variations, to maintain a light level slightly above the 300 lx threshold, to assess the performance of the VLC system and to explore in depth its energy efficiency potential.

Within these tests, the system is tested at 100 kb/s data rate, sufficient for transmitting emergency messages. Nevertheless, the prototype is compatible with data rates up to 500 kb/s, whereas higher data rates are achievable with an upgrade of the data processing unit. Therefore, the concept opens the path toward a fully functional Li-Fi system.

### 3.3. Experimental Results

**Figure 6:**
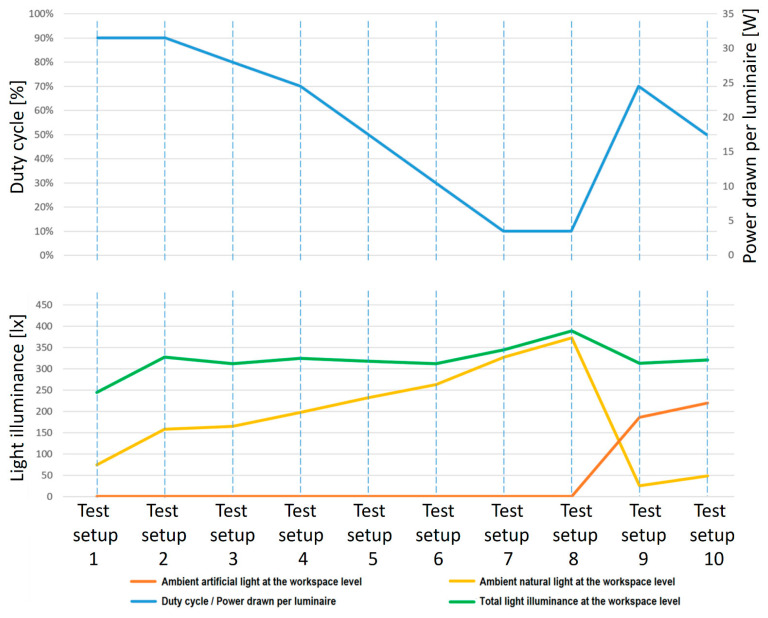
VLC prototype performance.

**Figure 7:**
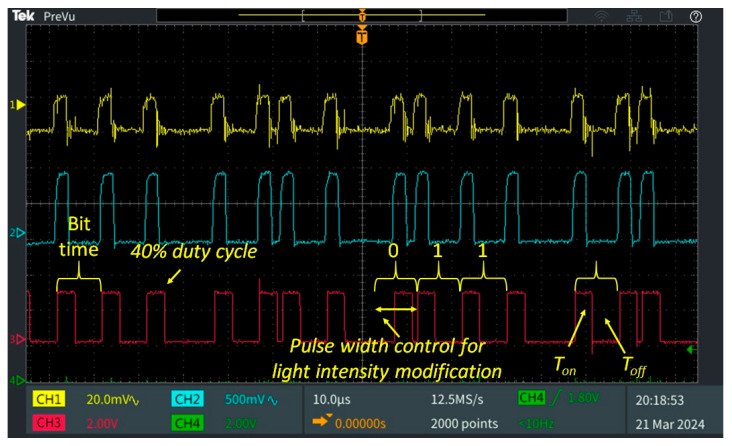
Oscilloscope screenshot displaying the VLC receiver signal processing for a frame modulated with a 40% duty cycle: Channel 1 shows the output from the transimpedance amplifier; Channel 2 presents the amplified signal; Channel 3 illustrates the output of the Schmitt trigger, which is used for data processing.

The synthesis of the experimental results is available in Figure 6, while Figure 7 presents an oscilloscope capture that shows the architecture of the signal processing chain at the VLC receiver unit. The data obtained from the ten testing setups highlight that the proposed prototype has the ability to autonomously analyze the lighting conditions located at the workstation level, to transmit this data through the dedicated optical channel and to regulate the light intensity by adjusting the duty cycle of the VLC transmitter.

Additionally, the experimental results reconfirmed the robustness of the proposed optical communication solution, the system demonstrating high transmission reliability even in low lighting regimes, maintaining a Bit Error Rate (BER) lower than the threshold of 10^−7^. This performance was evaluated by transmitting three distinct data sets of 10 million bits each for every testing setup, demonstrating the consistency and reliability of the communication mechanism under variable lighting conditions.

The analysis of the experimental results highlights the ability of the proposed prototype to dynamically modulate its duty cycle to maintain the illuminance above the specified minimum threshold of 300 lx. However, under low ambient lighting conditions, as shown in the first test, the limited emission capacity of the four LED tubes does not allow reaching the required value, stopping at a value of around 250 lx. This suggests the need to integrate additional lighting fixtures to ensure the desired level of illuminance, as demonstrated in Tests 9 and 10, where even under low ambient natural light, the contribution of adjacent luminaires helped achieve the desired illuminance of around 300 lx. In Tests 2 to 6, the natural light was enough to support the luminaire in reaching the required illuminance at the workspace level. In contrast, when the incident natural light is sufficient to ensure a properly illuminated work environment (Tests 7 and 8), the VLC system operates with a minimum duty cycle, generating only the light intensity indispensable for maintaining wireless optical communication. In such scenarios, the power consumption is reduced to almost negligible levels, in this case down to 3.6 W. For comparison, a standard broadband router, designed to provide wireless connectivity to user devices, has a power consumption in the range of 30–50 W, which is a significant difference in terms of energy efficiency. Furthermore, the duty cycle of the VLC transmitter can be further reduced to values of approximately 1% or even below [57,58]. In such circumstances, the user perceives the lighting source as being off, while the power consumption decreases proportionally to the reduction in the transmitter activation time, thus highlighting the potential of the system for ultra-low power applications.

A key aspect highlighted by the experimental evaluation is the prototype’s ability to maintain the stability of the active communication link, while simultaneously providing a BER lower than 10^−7^. This remarkable performance is attributable to a particularly favorable Signal-to-Noise Ratio (SNR), specific to the indoor testing environment, as well as to a rigorously optimized signal processing architecture integrated at the VLC receiver level.

## 4. Investigating the Visible Light Communications Concept’s Scalability and Its Potential for Energy Savings

### 4.1. Simulation Setup

The purpose of the following simulations is to demonstrate the capability to implement new features for indoor VLC systems, namely, efficient energy management and BMS integration. Thus, the following sections are aiming to show that the prototype in particular, and most important, the VLC technology in general, is able to adapt the duty-cycle of the VLC optical signal to improve the user experience, while significantly optimizing energy consumption.

**Figure 8:**
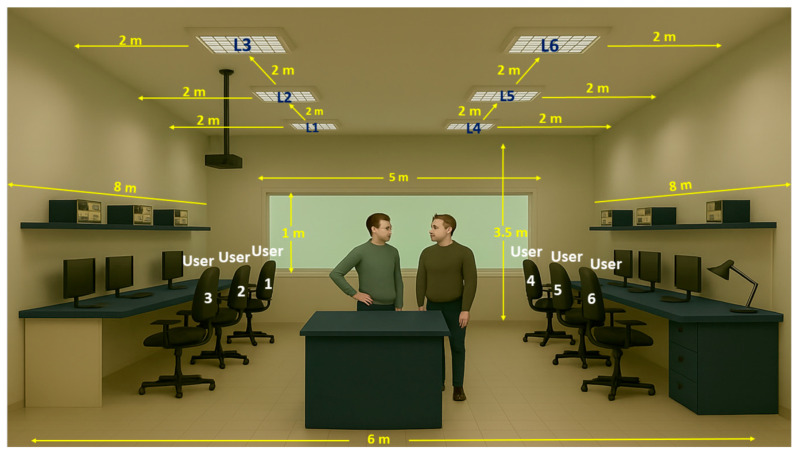
Illustration of the setup considered in the evaluation of the smart lighting system.

For this purpose, the prototype’s hardware features (presented in Section 3) have been integrated within a simulation model (presented in Section 2) which will be used to investigate its scalability and benefits. As illustrated in Figure 8, the simulation setup envisions an open-space co-working area, designed for 6 users, each user having their own lighting luminaire located slightly above the personal workspace. It is assumed that the work is carried out under daytime conditions with natural light entering through the windows, and also during night time. Therefore, to evaluate the system’s ability to adapt to different conditions, the simulation considers four different daylighting conditions, including a relatively sunny day, a day with variable sky partially covered by clouds, a cloudy day, and a night scenario assuming the absence of natural light. The purpose of these scenarios is to evaluate whether the concept is able to properly adapt to variable ambient lighting conditions to maintain an illumination level of around 300 lx at each user workspace. An additional aim is to estimate the increase in efficiency in the case of implementing an intelligent lighting system.

The workspace considered in this assessment has dimensions of 8 m in length, 6 m in width and 3.5 m in height. On one of the 6 m sides, a window with dimensions of 5 m in width and 1 m in height is located, which allows natural light to enter the interior. The lighting level relevant to activities in the co-working space is measured at a height of 0.8 m from the floor, corresponding to the workspace level. The scenario assumes that up to six people are usually present in this workspace, but their presence is not constant, which can influence the requirements for artificial lighting. To ensure adequate lighting, six artificial lighting fixtures are considered in the room. These are evenly spaced along the two long sides, three luminaires on each side, with a distance of approximately 2 m between them. This configuration aims to provide a uniform distribution of light on the work surface, while adapting to the variations in natural light coming through the window. Therefore, this setup is rather similar with the laboratory used for the experiments from Section 3. This setup allows the simulation and evaluation of how natural and artificial light combine to maintain an optimal level of lighting in the laboratory, taking into account the variable presence of people and external lighting conditions. On the other hand, we reemphasize that within this setup, energy efficiency should be obtained without affecting user comfort whatsoever. The described configuration is illustrated in Figure 8, while the setup parameters are summarized in Table 2.

### 4.2. Simulation Use Cases

#### 4.2.1. Analysis of the Distribution of Natural Light Coming from External Sources

The main purpose of this case study is to evaluate the impact of daylight on the distribution of lighting in the analyzed workspace. In particular, it aims to understand how daylight contributes to the general illumination of the room, influencing the comfort of users. Given that, in many situations, the natural light entering through windows can be sufficient for a large part of the users and/or for the efficient performance of most work tasks, the use of this natural resource brings multiple benefits. Among these are the reduction in energy consumption by reducing the need for artificial lighting, which leads to significant savings and a reduced environmental impact.

On the other hand, an insufficiently lit workspace can negatively affect the performance and comfort of users, leading to decreased productivity and increased levels of visual fatigue [60]. In addition, the lack of adequate natural lighting can determine the need for over-compensation through excessive artificial lighting, which contradicts the principles of energy efficiency and can generate additional costs [61,62]. Thus, a correct and detailed assessment of the distribution of natural light in the analyzed space is essential to optimize both working conditions and energy consumption. By adopting appropriate daylighting design and management strategies, comfortable, efficient and sustainable working environments can be created that meet the needs of users and maximize the benefits offered by natural light.

Therefore, the purpose of this investigation is to provide an assessment regarding the impact of the natural light within the lighting requirements and lighting distribution within the considered workspace. Evaluation of the lighting level in the laboratory according to the intensity of the natural outdoor light under various daylight conditions, ranging from a relatively bright day at 20,000 lx, to an average daylight at 10,000 lx, a cloudy day at 5000 lx, and a night scenario. Thus, the aim of this analysis is to identify the need to use additional artificial lighting to maintain optimal lighting, of at least 300 lx at the level of the workspace.

For this evaluation, a spatial light distribution model was used (described in Section 2), which takes into account characteristics of the natural light, as well as the conditions of the room. The model simulates the propagation and attenuation of the light flux at different measurement points in the working space area, providing an estimate of the level of natural lighting available indoors for each outdoor lighting scenario. Based on the model described, a Python 3 script was developed that automates the simulation process. The script considers the intensity of the outdoor natural light as an input signal and calculates the effective natural lighting in the laboratory according to the spatial distribution and characteristics of the laboratory. The representation of the adaptive dimming control algorithm is illustrated in Figure 3. The proposed algorithm dynamically adjusts the duty cycle based on feedback received via IR communication from the workspace level. The adjustment process is designed as a closed-loop control system, ensuring a real time response to actual illumination conditions rather than relying on preset values. This feedback mechanism inherently provides fine-tuning, as the algorithm continuously compares the adjusted results against the desired lighting state and makes corrections accordingly. In terms of response speed and stability, the algorithm was developed to achieve rapid convergence while avoiding oscillations. The feedback update interval is sufficiently low in order to avoid noticeable or perceivable flickering. Furthermore, the preliminary experimental validation detailed in Section 3 demonstrated that the system responds promptly to changes in workspace illumination while maintaining stable operation.

**Figure 9:**
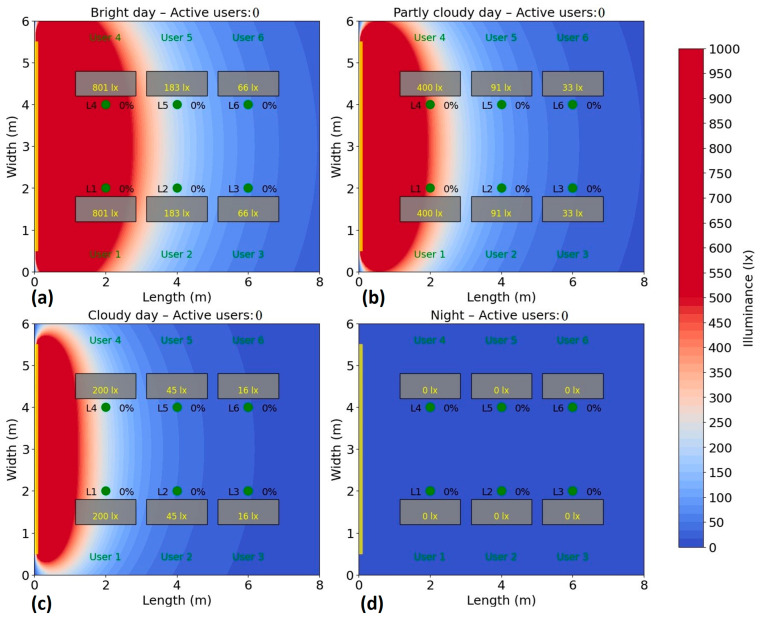
Simulation results showing how natural light is dispersed through the left side positioned window within the considered test area: (**a**) Bright day; (**b**) Partly cloudy day; (**c**) Cloudy day; (**d**) Night time.

The simulation results are presented in Figure 9 and show that on bright days with average sunshine, natural lighting makes a major contribution, eliminating the need for artificial lighting. Thus, on sunny days, the light entering through the window varies between 66 and over 800 lx at the workspace level, with the use of artificial lighting not being necessary for only 2 out of 6 workspaces. That would mean that four users might decide to switch on the light, leading to a very inefficient use of lighting system.

On days with average sunlight, the light varies between approximately 33 and 400 lx at the workspace level, meaning that, again, four users may need additional artificial lighting. On the other hand, on cloudy days, artificial lighting will be necessary for every user to maintain the target level of at least 300 lx at the workspaces, but not every one of them would need the same amount of artificial light.

#### 4.2.2. Assessing the Benefits of Adapting Artificial Light to the Presence of Users and the Influence of Neighboring Light Sources

The second evaluation assesses the system’s ability to automatically adjust artificial lighting based on available natural light, maintaining an illumination level of at least 300 lx while minimizing energy consumption and maintaining user comfort. Based on the model described in Section 2, the Python script was extended with a routine for artificial lighting adjustment under the constraint of keeping the total energy consumption at minimum. The script considers the intensity of the outdoor natural light as before, and, subsequently, dynamically adjusts the duty cycle factor of the artificial sources to compensate for variations in natural lighting and maintain the total lighting level close to the target value of at least 300 lx only for occupied places. This approach allows for automatic analysis of the lighting system’s behavior under varying conditions, clearly identifying compensation thresholds and the effectiveness of adjustments. In addition, the script provides the ability to generate reports on the required duty cycle and deviation from target illumination for each scenario, thus facilitating continuous system optimization and informed decisions on the need to install additional artificial lighting. Therefore, according to the implemented algorithm, the system adjusts the intensity of artificial sources in real time, adjusting the dimming for the necessary lights.

The scenario simulates the variation in the presence of users, each being randomly assigned to a workstation, and the related light source being automatically activated. The distribution of light is analyzed according to this configuration, following the influence of the lighting of the occupied stations on the neighboring ones. In the proposed lighting algorithm, optimal lighting is considered when every user experiences at least 300 lx at the workspace level, while minimizing the total energy consumption across the room. This is accomplished by adjusting the duty cycle of each luminaire accordingly. Thus, the proposed algorithm delivers the required light, not strictly from a single luminaire but with some contributions from the neighboring luminaires.

**Figure 10:**
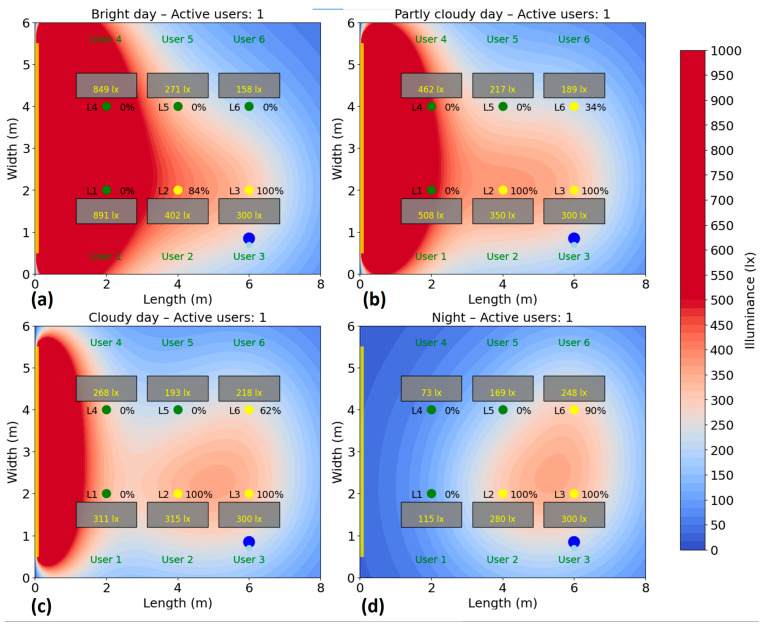
Simulation showing the dynamic response of the lighting system in the presence of one user, rep-resented by two blue dots (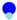): (**a**) Bright day; (**b**) Partly cloudy day; (**c**) Cloudy day; (**d**) Night time.

**Figure 11:**
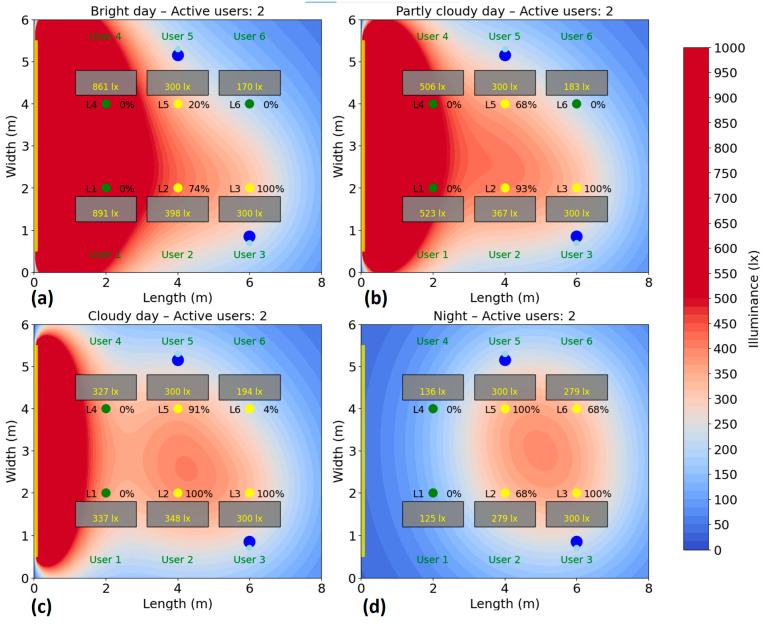
Simulation showing the dynamic response of the lighting system in the presence of two users, each user being represented by two blue dots (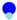): (**a**) Bright day; (**b**) Partly cloudy day; (**c**) Cloudy day; (**d**) Night time.

**Figure 12:**
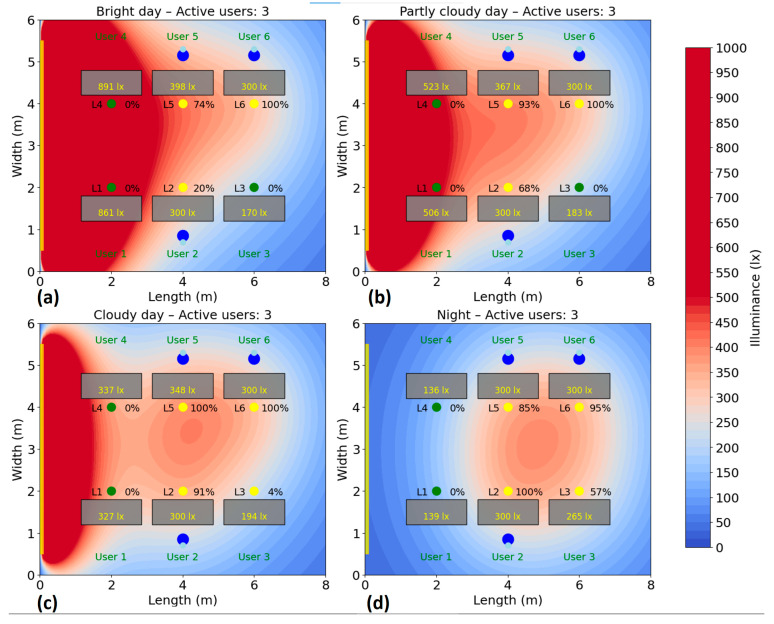
Simulation showing the dynamic response of the lighting system in the presence of three users, each user being represented by two blue dots (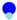): (**a**) Bright day; (**b**) Partly cloudy day; (**c**) Cloudy day; (**d**) Night time.

**Figure 13:**
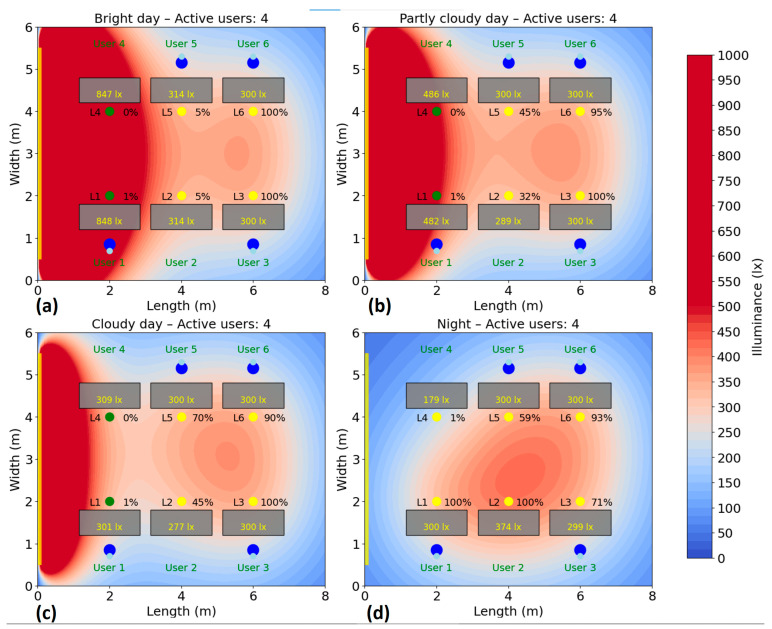
Simulation showing the dynamic response of the lighting system in the presence of four users, each user being represented by two blue dots (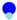): (**a**) Bright day; (**b**) Partly cloudy day; (**c**) Cloudy day; (**d**) Night time.

**Figure 14:**
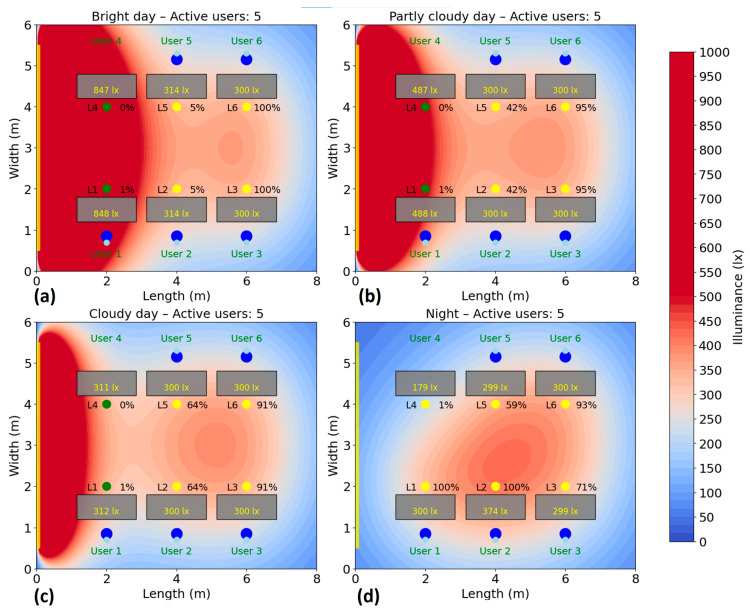
Simulation showing the dynamic response of the lighting system in the presence of five users, each user being represented by two blue dots (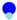): (**a**) Bright day; (**b**) Partly cloudy day; (**c**) Cloudy day; (**d**) Night time.

**Figure 15:**
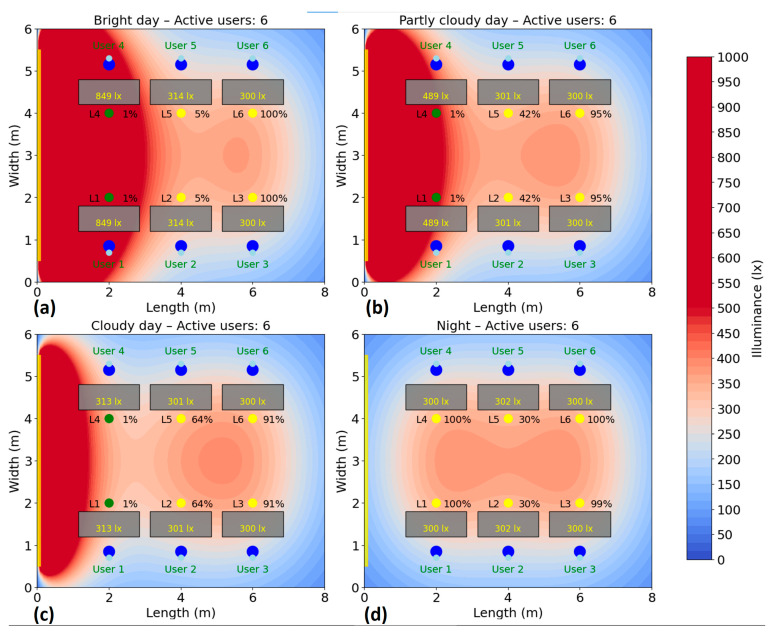
Simulation showing the dynamic response of the lighting system in the presence of six users, each user being represented by two blue dots (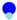): (**a**) Bright day; (**b**) Partly cloudy day; (**c**) Cloudy day; (**d**) Night time.

The test is repeated for several scenarios, highlighting the behavior of the system and its efficiency according to the presence, without compromising user comfort. Energy consumption is monitored and recorded during the test period to calculate the savings achieved compared to a fixed system. The simulation of the energy consumption for the automatic lighting system was performed for six different scenarios, corresponding to a number of 1–6 active users in the area. In each case, the users were randomly distributed to their places, and the consumption was analyzed under four natural lighting conditions: bright day (20,000 lux), average day (10,000 lux), cloudy day (5000 lux) and night (0 lux), the results being presented in Figure 10, Figure 11, Figure 12, Figure 13, Figure 14 and Figure 15.

In the single-user scenario (Figure 10), the intelligent lighting system provides the necessary lighting only in the active user area, maintaining a low energy consumption. Artificial lighting is dynamically adapted according to the level of natural light and the user’s choice, using selective lighting fixtures L3, together with neighboring ones (L2, and L6), if necessary. Since the single use of L3 is not sufficient to ensure the minimum lighting level, neighboring lighting fixtures are used at different intensities, adapted in order to compensate the natural lighting level. Thus, in bright day conditions, the total consumption is at a ≈31% level, whereas in partially and fully cloudy conditions the consumption is at ≈39% and ≈44%, respectively. At night, lighting is also provided locally, with a consumption of ≈48% of the maximum intensity. Therefore, the system accurately identifies the user’s position and activates only the lighting needed for that area, maximizing energy efficiency without compromising visual comfort.

In the case with two users (Figure 11), the consumption slightly increases. During bright daylight, consumption is approximately 70 W (32%), in average natural light conditions it rises to 95 W (44%), and on cloudy days it approaches 106 W (49%). In the complete absence of natural light (at night), total consumption is approximately 121 W, representing 56% of the total available power. The increase is only slightly higher than in the single-user scenario, as the system already used up to three lighting fixtures to ensure optimal illuminance.

With three users present (Figure 12), the system continues to use three or four lighting fixtures, adjusting the artificial light in accordance with the proximity of each one to the windows and the level of natural lighting. In favorable conditions (bright daylight), consumption is 70 W (32%). On an average day, consumption rises to 95 W (44%), and on a cloudy day it reaches 105 W (49%). In the night scenario, consumption is 123 W, representing 57% of the total capacity. Compared to the single-user scenario, where the system used up to three fixtures to ensure adequate illuminance, the increase in energy consumption with three users’ rests moderate. This minimal growth in energy use, despite the rising number of users, highlights the system’s ability to scale lighting intelligently, activating only the necessary fixtures and adjusting their intensity based on each user’s position and ambient light availability.

For the four-user scenario (Figure 13), energy consumption remains relatively low under strong natural light conditions, recording 76 W (35% of the total). As the natural light decreases, the system increases the artificial light output: consumption reaches 99 W (46%) on an average day and 110 W (51%) on a cloudy day. At night, consumption reaches 153 W, i.e., 71% of the maximum capacity of the 6 luminaires. It is important to note that the system does not unnecessarily switch on all luminaires, activating only those that effectively contribute to the required illumination of the occupied areas. Another observation is that the luminaire of user 1 is at 1% intensity even when they have sufficient natural light, maintaining VLC connectivity this way. Another important aspect is that the algorithm activated lighting fixture L2 even though no user was directly beneath it because, due to its central positioning, L2 effectively contributed to illuminating areas for users 1, 3, 5, and 6, enhancing overall energy efficiency.

In the case of five active users (Figure 14), consumption increases moderately compared to the first three scenarios, yet remains comparable to the 4-user scenario. On a bright day, consumption is 76 W (35%), reflecting the ongoing use of selective lighting based on users’ presence and natural light availability. In scenarios with medium and low natural light, the total consumption reaches 99 W (46%) and 112 W (52%), respectively. During the night, the necessary consumption reaches 153 W, which represents 71% of the total power available. Thus, compared to the case with four users, the algorithm maintains the same energy consumption, despite the extra user. This is achieved by efficiently sharing light from nearby fixtures, avoiding the need to activate additional ones.

In the scenario with six users (Figure 15), i.e., full workspace occupancy, the increase in energy consumption is minimal and not at its maximum across all conditions. On a bright day, an average day, and a cloudy day, the consumption remains nearly identical to that in the five-user case: 78 W (36%), 99 W (46%), and 112 W (52%), respectively. In the complete absence of natural light, consumption rises slightly to 166 W (77%). Even under full occupancy, the system is able to avoid reaching maximum capacity (216 W), demonstrating its ability to manage lighting efficiently by distributing illumination where needed and minimizing unnecessary energy use. Thus, needless oversizing of consumption is avoided, and the natural light supply is used as efficiently as possible.

**Figure 16:**
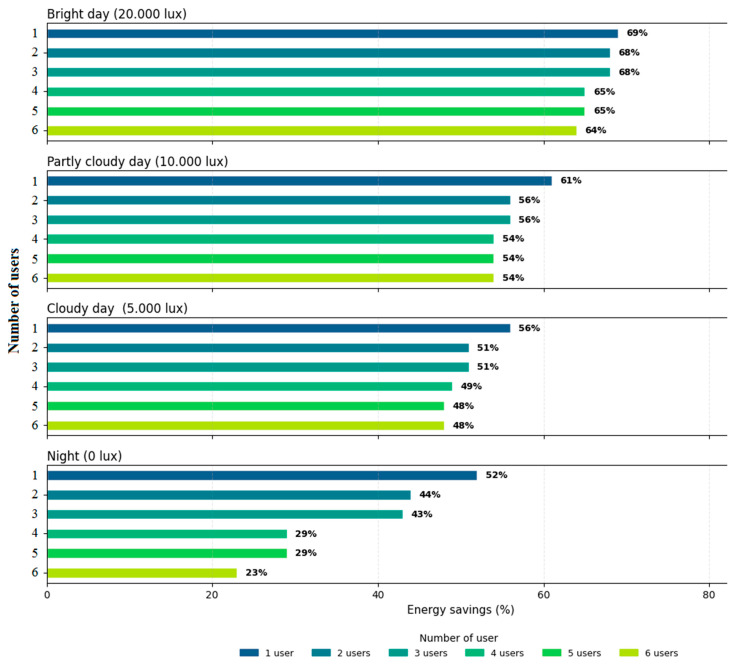
Summary of energy savings based on the number of active users, relative to the scenario in which all lighting fixtures operate at 100% capacity.

Figure 16 summarizes the energy savings achieved for the four natural lighting conditions, based on a random number of active users ranging from one to six. The behavior demonstrates intelligent control of resources, with direct benefits on energy savings and visual comfort of users. These results show that the lighting system manages energy efficiently, automatically scaling consumption according to the number of active users and the level of natural light available. Consumption remains significantly lower than that associated with a continuous use of lighting at 100%, which indicates a substantial saving in all tested scenarios.

## 5. Conclusions

In the context where energy efficiency and wireless communication technologies are major concerns of today’s society, the proposed indoor VLC prototype is able to ensure energy management in a much more efficient way. The proposed concept is implemented and experimentally evaluated under laboratory conditions. While the demonstrated data rate was limited at 100 kb/s with a BER under 10^−7^, the notable feature lies in its dynamic light dimming capability. Experimental results show that the developed prototype is able to ensure significant energy savings by integrating lighting management techniques, combined with a VLC emitter capable of adjusting its light intensity by dynamically changing the duty cycle. The results also demonstrate that the VLC prototype can provide optimal feedback, with response times that do not affect user comfort. The system proved to be able to automatically adjust the LED duty cycle between 10% and 90%, responding to ambient natural or artificial light to maintain a minimum workspace illuminance of 300 lx.

Furthermore, a scalability assessment was performed via simulations in an office setting accommodating up to six users. The results confirm the system’s ability to dynamically regulate lighting levels and sustain communication based on user presence, achieving energy savings between 23% and 69% without sacrificing visual comfort or connectivity quality. Simulation testing of the smart lighting system with VLC technology demonstrates extensive functionality, combining energy efficiency with innovations in data transmission. The system is able to maintain constant lighting under dynamic conditions, without significant fluctuations. Energy efficiency is significantly improved due to adaptive lighting adjustment.

Although the results are encouraging, it must be highlighted that the luminaires were idealized as Lambertian sources. In realistic indoor VLC setups, especially at short ranges, luminaires often include diffusers, collimators, reflectors, or other beam-shaping optics. These modify the emission pattern, meaning that actual luminaires deviate from the ideal Lambertian distribution used for the theoretical work. Nevertheless, the experimental results confirm that the Lambertian approximation is not far from reality: the deviations introduced by non-ideal optical components do affect performance, but not to an extent that invalidates the general conclusions. In addition, the non-ideal nature of the electronic components involved in designing the modulators can significantly affect the performance of the entire VLC chain. For example, transistor heating due to current flow, power losses in drivers, parasitic capacitances and resistances, and limitations in power supply design can lead to thermal drift, bandwidth reduction, nonlinear distortion, and limited efficiency. These effects often become more pronounced under high modulation frequencies, high drive currents, or long continuous operation. If unaccounted for, they can degrade the link quality, reduce signal-to-noise ratio, increase bit error rates, or limit achievable data rates.

Future research should focus on optimizing the hardware prototype and on additional experimental investigations in complex scenarios, reflecting real-life daily activities. It is recommended to expand the prototype for real-world testing under continuous use conditions and implement predictive algorithms to anticipate lighting changes, including compensation for the influence of neighbors. The expansion of the VLC function can be performed in classrooms, libraries and laboratories, with a focus on indoor guidance for visitors, transmission of course materials and/or real-time location of equipment.

Thanks to its adaptive light intensity control algorithm, this VLC solution holds significant promise for integration into smart indoor environments that prioritize sustainability alongside seamless connectivity.

## Figures and Tables

**Table 1:** Summary of Testing Parameters for the VLC Prototype.

VLC Prototype Functionalities	Characteristics
Optical Communication Parameters	-Visible light for download and infrared radiation for upload with full-duplex communication capability; -Automatic light intensity adjustment based on brightness measured at the VLC receiver; -Modified VPPM with duty cycle adjustable between 10% and 90%; -Data rate of 100 kb/s
Lighting Source Parameters	-VLC transmitters integrated into the lighting luminaire with 4 × 60 cm 9 W LED tubes; -Optical emission up to 188 lx at the workspace level (2.7 m from the ceiling) for each luminaire; -Infrared optical receiver for data reception, also used for feedback in illuminance adjustment; -Work room size: 8 m × 6 m × 3.5 m; -Lighting luminaire arranged in a 2 m × 2 m grid; -Workspaces located under each luminaire; -Window placed along the width of the room, measuring 5 m × 1 m;
Mobile VLC Device Parameters	±53° FoV; -Adaptive gain; -Data processing in real-time; -No error correcting codes; -Illuminance measurement function; -Data upload function using an IR transmitter.

**Table 2:** Summary of test area characteristics.

Envisioned Aspect	Details
Room dimensions	8 m (length) × 6 m (width) × 3.5 m (height)
Workspace level	0.8 m from the floor
Window	5 m × 1 m (on the 6 m side)
Maximum number of people	6; Variable presence of people, not all simultaneously;
Lighting fixtures	6 × 36 W; Light fixture arrangement: 3 on each 8 m side; The fixtures are placed on a 2 m × 2 m grid
Lighting purpose	VLC coverage; Uniform distribution; Natural light compensation; Maintain user(s) comfort

## Data Availability

The original contributions presented in this study are included in the article. Further inquiries can be directed to the corresponding author(s).

## Abbreviations

The following abbreviations are used in this manuscript:

VLC	Visible Light Communications
Li-Fi	Light-Fidelity
BER	Bit Error Rate
EU	European Union
EPBD	Energy Performance of Buildings Directive
IEA	International Energy Agency
EED	Energy Efficiency Directive
BMS	Building Management System
ETS	Emissions Trading Scheme
ZEB	Zero Emissions Buildings
FoV	Field-of-View
JRC	Joint Research Centre
BIPV	Building Integrated Photovoltaics
EMI	ElectroMagnetic Interference
VPPM	Variable Pulse Position Modulation
PWM	Pulse Width Modulation
PPM	Pulse Position Modulation
IR	InfraRed
AGC	Automatic Gain Control
SNR	Signal-to-Noise Ratio
